# Source identification and driving factor apportionment for soil potentially toxic elements via combining APCS-MLR, UNMIX, PMF and GDM

**DOI:** 10.1038/s41598-024-58673-9

**Published:** 2024-05-13

**Authors:** Cang Gong, Xiang Xia, Mingguo Lan, Youchang Shi, Haichuan Lu, Shunxiang Wang, Ying Chen

**Affiliations:** 1 Research Center of Applied Geology of China Geological Survey, Chengdu, China; 2 Key Laboratory of Natural Resource Coupling Process and Effects, Beijing, China; 3 Technology Innovation Center for Analysis and Detection of the Elemental Speciation and Emerging Contaminants, China Geological Survey, Kunming, China

**Keywords:** Potentially toxic elements, Source identification, APCS-MLR, UNMIX, PMF, Geographic detector, Environmental sciences, Environmental social sciences

## Abstract

The contamination and quantification of soil potentially toxic elements (PTEs) contamination sources and the determination of driving factors are the premise of soil contamination control. In our study, 788 soil samples from the National Agricultural Park in Chengdu, Sichuan Province were used to evaluate the contamination degree of soil PTEs by pollution factors and pollution load index. The source identification of soil PTEs was performed using positive matrix decomposition (PMF), edge analysis (UNMIX) and absolute principal component score-multiple line regression (APCS-MLR). The geo-detector method (GDM) was used to analysis drivers of soil PTEs pollution sources to help interpret pollution sources derived from receptor models. Result shows that soil Cu, Pb, Zn, Cr, Ni, Cd, As and Hg average content were 35.2, 32.3, 108.9, 91.9, 37.1, 0.22, 9.76 and 0.15 mg/kg in this study area. Except for As, all are higher than the corresponding soil background values in Sichuan Province. The best performance of APCS-MLR was determined by comparison, and APCS-MLR was considered as the preferred receptor model for soil PTEs source distribution in the study area. ACPS-MLR results showed that 82.70% of Cu, 61.6% of Pb, 75.3% of Zn, 91.9% of Cr and 89.4% of Ni came from traffic-industrial emission sources, 60.9% of Hg came from domestic-transportation emission sources, 57.7% of Cd came from agricultural sources, and 89.5% of As came from natural sources. The GDM results showed that distance from first grade highway, population, land utilization and total potassium (TK) content were the main driving factors affecting these four sources, with q values of 0.064, 0.048, 0.069 and 0.058, respectively. The results can provide reference for reducing PTEs contamination in farmland soil.

## Introduction

After years of urbanization and industrialization, soil potentially toxic elements (PTEPTEs) pollution caused by high-intensity and intensive human activities has become a global environmental problem^1–6^. It changes the chemical and physical properties of soil, reduces the quality of soil and crops, and further endangers human health through the food chain^1,7–9^. In China, the pollution of PTEs in soil is more prominent in some areas. According to the National soil pollution Survey^10^, the over-standard rates of Cd, Hg, As, Cu, Pb, Cr, Zn and Ni were 7.0%, 1.6%, 2.7%, 2.1%, 1.5%, 1.1%, 0.9% and 4.8%, respectively. Therefore, taking measures to control and repair PTEs contamination in farmland soil is the key to ensure soil environment quality and agricultural products safety.

Quantitative research on the source analysis and driving factors of soil PTEs is of great significance for effective control of soil PTEs contamination. In general, soil PTEs sources are influenced by natural factors, anthropogenic activities factors or a combination of these factors^3,10–13^. Anthropogenic factors include mining activities, agricultural activities, industrial activities, transportation activities, etc., while natural factors include soil parent material, elevation, hydrological conditions, etc. The combined action of these factors further leads to the complex spatial variability of PTEs in soil, which increases the difficulty of source analysis.

Quantitative source analysis of PTEs is to use the chemical and physical characteristics of contaminants in receptor model to distinguish contamination sources and quantify contribution apportionment rate of various sources. Among them, positive matrix decomposition (PMF), edge analysis (UNMIX) and absolute principal component scores–multiple line regression (APCS-MLR) were frequently used in soil PTEs source analysis^14–16^. APCS-MLR obtains the absolute factor score and the quantitative contribution of each factor by reducing and multiple linear regression respectively^15^. UNMIX model automatically delete unreasonable data through the system model, does not need to set contamination sources number and uncertainty, and reduces anthropogenic factors influence^16^. According to the concentration of PTE in each soil sample, PMF obtained the source profile, source contribution and the corresponding uncertainty^14^. Due to the different algorithms used, the results of source distribution of PTEs in soil by these receptor models may be different. As these models require numerical data to satisfy the algorithm, type variables such as soil parent material and land use type cannot be calculated^16^. In addition, the spatial variability of data was not taken into account by these models. In fact, spatial stratification heterogeneity among variables has significant effect on the accumulation of soil PTEs.

In contrast, the geo-detector method (GDM) can well overcome the limitations of PMF, UNMIX and APCS-MLR models. GDM is an effective tool for spatial variance analysis, which can detect the nonlinear relationship among multiple explanatory variables, quantitatively determine the influence of various influencing factors on the spatial heterogeneity of soil PTEs, and solve the limitations of traditional methods in analyzing category variables^17–19^. It has been widely used in groundwater^20^, land use^21,22^, ecological vulnerability^23,24^, soil PTEs pollution^10,25, 26^ and other fields. Therefore, based on measurement data, auxiliary data and spatial information, this study proposes a comprehensive method to combine receptor models APCS-MLR, UNMIX and PMF with GDM. Auxiliary data include classification variables, such as elevation, slope, aspect, land use type, soil pH, organic matter (OM), total nitrogen (TN) content, total phosphorus (TP) content, total potassium (TK) content. Spatial information includes distance from industrial land, commercial distance, railway distance, residential land distance, highway distance and river distance. This approach provides an in-depth understanding of the source analysis and driving factors identification of PTEs in soil at the town scale.

The study area is located in the heart of Chengdu Plain, Sichuan Province, which is a famous national agricultural park and Chinese chives production base. However, with the rapid expansion of urbanization, the rapid development of industrial enterprises and the widespread use of agricultural substances such as pesticides and chemical fertilizers, the soil environment of Chengdu Plain has changed to varying degrees, directly affecting the content of PTES in agricultural products in Chengdu Plain. In this study, it was speculated that anthropogenic factors such as traffic activities, industrial activities and agricultural activities may be the main pollution sources of HMs in the soil of the study area. Combined with the spatial information and spatial analysis data of the sampling points, this hypothesis was tested by a comprehensive method, and the source analysis of PTEs in the soil was objectively explained. The purpose of this study was to (1) evaluate the level of PTEs contamination in the central soil of Chengdu Plain, Sichuan Province; (2) APCS-MLR, UNMIX and PMF models were used to assign potential contamination sources of soil PTEs; (3) GDM was used to analysis the driving factors affecting soil PTEs pollution sources. The research results revealed the potential contamination sources and corresponding driving factors of soil PTEs contamination, and provided targeted strategies for the control of soil PTEs contamination.

## Materials and methods

### Study area

The study area is located in the central zone of Chengdu Plain, Chengdu, Sichuan Province, about 40 km away from the urban area of Chengdu. The study area covers an area of 80 km^2^ and a population of 7.51 × 10^4^. Except a small part of the area is shallow hill platform, most of the area is flat dam, and 90% of the soil is black oil-sand soil formed by impact. 3/4 of the research area is located in the water source protection area, and many major rivers such as Baitiao river and Xuyan river flow through this area. Major transportation hubs such as National highway 317, Chengguan expressway and Chengguan express railway run through the whole territory. The land use type is mainly cultivated land (about 46.5%), followed by forest land (24.1%) and residential land (13.9%), and 1.9% of industrial land is scattered in the southwest and central regions.

### Sample collection and measurement

**Figure 1:**
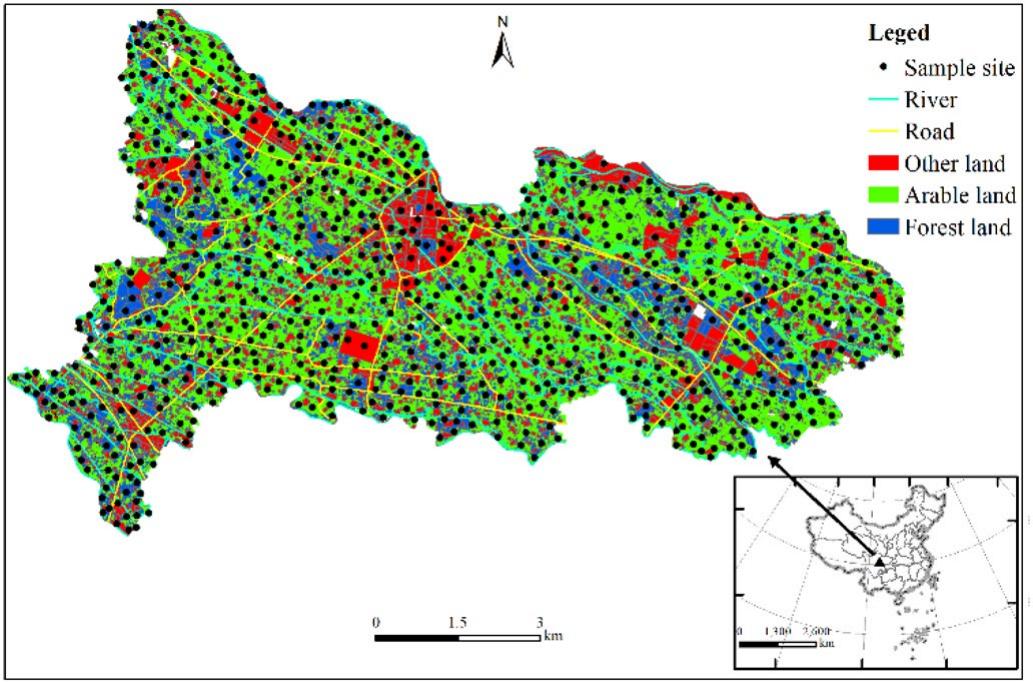
Distribution of sampling locations in the study area (map were generated with software ArcMap10.8 http://www.esri.com/).

The sample sites were arranged in 1 km^2^ grid, the density of cultivated land and forest land was 9 points km^−2^, and the density of residential areas, schools, industrial land and construction land was 4 points km^−2^. Sample collection will be completed in April 2021. According to the Code for Geochemical Evaluation of Land Quality (DZ/T0295-2016), 788 samples of topsoil (0–20 cm) were collected (538 samples of cultivated soil, 209 samples of forest soil and 41 samples of other land soil). The sampling locations were recorded by GPS. The sampling location is shown in Fig. 1. In order to improve sample representativeness, the "X" sampling method (1 sub-sample was collected at each of the 4 end points of "X" and the middle crossing point) was used to collect 5 sub-samples from a 20–50 m area around each sampling point and mix them into one sample. The samples were air-dried for a week, removed from debris, passed through a 10-mesh plastic sieve, and sent to the Institute of Comprehensive Utilization of Mineral Resources, Chinese Academy of Geological Sciences for analysis and testing. pH was determined by ion selective electrode method (for water extraction without carbon dioxide, the ratio of soil to water was 1/2.5), TOC by volumetric method (analysis methods for regional geochemical sample-part 27: determination of organic carbon contents by potassium dichromate volumetric method (DZ/T 0279. 27–2016)), TN by combustion infrared method, As, Hg and Se by atomic fluorescence method, Cu, Pb, Zn, Ni, Cr, Cd, TP and TK by X-ray fluorescence method and inductively coupled plasma optical /mass spectrometry. The quality of analysis and test was controlled by means of inserting national soil standard substances (GBW07424, GBW07425, GBW07426, GBW07427 and GBW07428), repeatability inspection, anomaly inspection and blank test.

### Pollution assessment

The pollution factor (PF) and pollution load index (PLI) were used to evaluate the pollution degree of soil PTEs^10,16, 21^. PF and PLI were calculated based on the Eqs. (1), (2):

1 $${\text{PF}}=\frac{{C}_{i}}{{C}_{b}}$$

2 $${\text{PLI}}=\sqrt[n]{{PF}_{1}\times {PF}_{2}\times \cdots .\times {PF}_{n}}$$

where C_i_ is the concentration of soil PTEs, C_b_ is the geochemical background concentration of Sichuan province. There are five classes based on PF value: ≤ 1 (uncontaminated), 1 < PF ≤ 2 (slightly contaminated), 2 < PF ≤ 3 (mild contaminated), 3 < PF ≤ 5 (moderate contaminated), > 5 (high contaminated). Four classes based on PLI value: ≤ 1 (uncontaminated), 1 < PLI ≤ 2 (moderate contaminated), 2 < PLI ≤ 5 (highly contaminated), > 5 (extremely contaminated).

### Absolute principal component scores–multivariate linear regression (APCS-MLR)

The APCS-MLR model was proposed by Thurston and Spengler in 1985. Which can determine the load of PTEs to each pollution source, and calculate the average contribution of each source to soil PTEs. The detailed steps are as follows:

3 $${Z}_{ij}=\frac{{C}_{ij}-\overline{C}}{{\sigma }_{i}}$$

4 $${{(Z}_{0})}_{i}=\frac{0-\overline{{C }_{i}}}{{\sigma }_{i}}=-\frac{\overline{{C }_{i}}}{{\sigma }_{i}}$$

5 $${{\text{X}}}_{{\text{i}}}={{\text{b}}}_{0}+\sum_{{\text{k}}=1}^{{\text{m}}}{{\text{b}}}_{{\text{k}}}{{\text{APCS}}}_{{\text{k}}}$$

where Z_ij_ is the concentration after normalization, C_ij_ is the concentrations of the *i*th sample of the *j*th PTE, $\overline{{\text{c}} }$ is the average concentrations of* j*th PTE, ${\upsigma }_{{\text{i}}}$ is the standard deviation of the *j*th PTE, b_0_ is the intercept of regression for soil PTE, b_k_ is the regression coefficient of the soil PTE, m is the number of factors, APCS_k_ is the adjusted score of the* k*th source, b_k_APCS_k_ can be regarded as the contribution of the *k*th source to the soil PTE concentrations.

### UNMIX model

In this model, the data space dimensionality is reduced via singular value decomposition, and then number of sources, source composition and contribution rate of sources of each sample can be estimated (USEPA, 2007). The fundamental model can be characterized as follow:

6 $${C}_{ij}=\sum_{k=1}^{m}{U}_{ik}{D}_{kj}+{S}_{ij}$$

where C_ij_ is the concentrations of *b*th PTEs in *i*th sample, U_ik_ is the contribution of *k*th in the *i*th sample, D_kj_ is the concentrations of the *j*th PTEs from *k*th source, S_ij_ is the error. The source component spectrum parsed by the model needs to meet minimum system requirements that can be interpreted by the model (Min Rsq(R^2^) > 0.8, Min Sig/Noise(S/N) > 2).

### Positive Matrix Factorization (PMF)

The PMF model decomposed the original matrix X_ij_ into two factor matrices g_ik_ and f_kj_ as well as a residual matrix e_ij_, and it was expressed as follows (USEPA, 2014):

7 $$x_{ij} = \mathop \sum \limits_{k = 1}^{p} g_{ik} f_{kj} + e_{ij} x_{ij} = \mathop \sum \limits_{k = 1}^{p} g_{ik} f_{kj} + e_{ij}$$

where, *x*_*i*j_ is the concentration of the* j*th PTE in the *i*th sample (mg/kg); *g*_*ik*_ is the contribution of the* k*th source in the *i*th sample; *f*_*kj*_ is the concentrations of the *j*th PTE from the *k*th source factor; and *e*_*ij*_ is the residual. The residual error matrix e_ij_ is calculated by the minimum value of the objective function Q calculated according to the Eq. (9):

8 $${\text{Q}}=\sum_{i=1}^{n}\sum_{j=1}^{m}{\left[\frac{{e}_{ij}}{{u}_{ij}}\right]}^{2}$$

where u_ij_ is the uncertainty of the *j*th PTEs in *i*th samples. The uncertainty (u) of the PTEs was calculated as follows^27^:

When the concentration values below the detection limit:

9 $$x_{ij} = \frac{{d_{ij} }}{2},\,u_{ij} = \frac{{d_{ij} }}{2}$$

When the concentration values beyond the detection limits:

10 $${x}_{ij}={c}_{ij}$$

11 $${\text{if}}\, x_{ij} \le \,{3}\,d_{ij} ,\,u_{ij} = \frac{{d_{ij} }}{3} + 0.2 \times c_{ij}$$

12 $${\text{if}}\,x_{ij} > \,3\,d_{ij} ,\,u_{ij} = \frac{{d_{ij} }}{3} + 0.1 \times c_{ij}$$

For missing values:

13 $$x_{ij} = \overline{{c_{ij} }} \,u_{ij} = 4\overline{{c_{ij} }}$$

where x_ij_ is the concentration of sample species, d_ij_ is the detection limit, σ_ij_ is the x_ij_ concentration uncertainty, c_ij_ is the sample measured concentration, $\overline{{c }_{ij}}$ is the measured concentrations geometric mean.

### Geo-detector method (GDM)

GDM was an effective tool to analyze the spatial variance that can identify the explanatory variables affecting the dependent variable based on the assumption that explanatory variable (X) is associated with dependent variable (Y) if their spatial pattern is consistent^17^. The detailed calculation references^17^.

**Figure 2:**
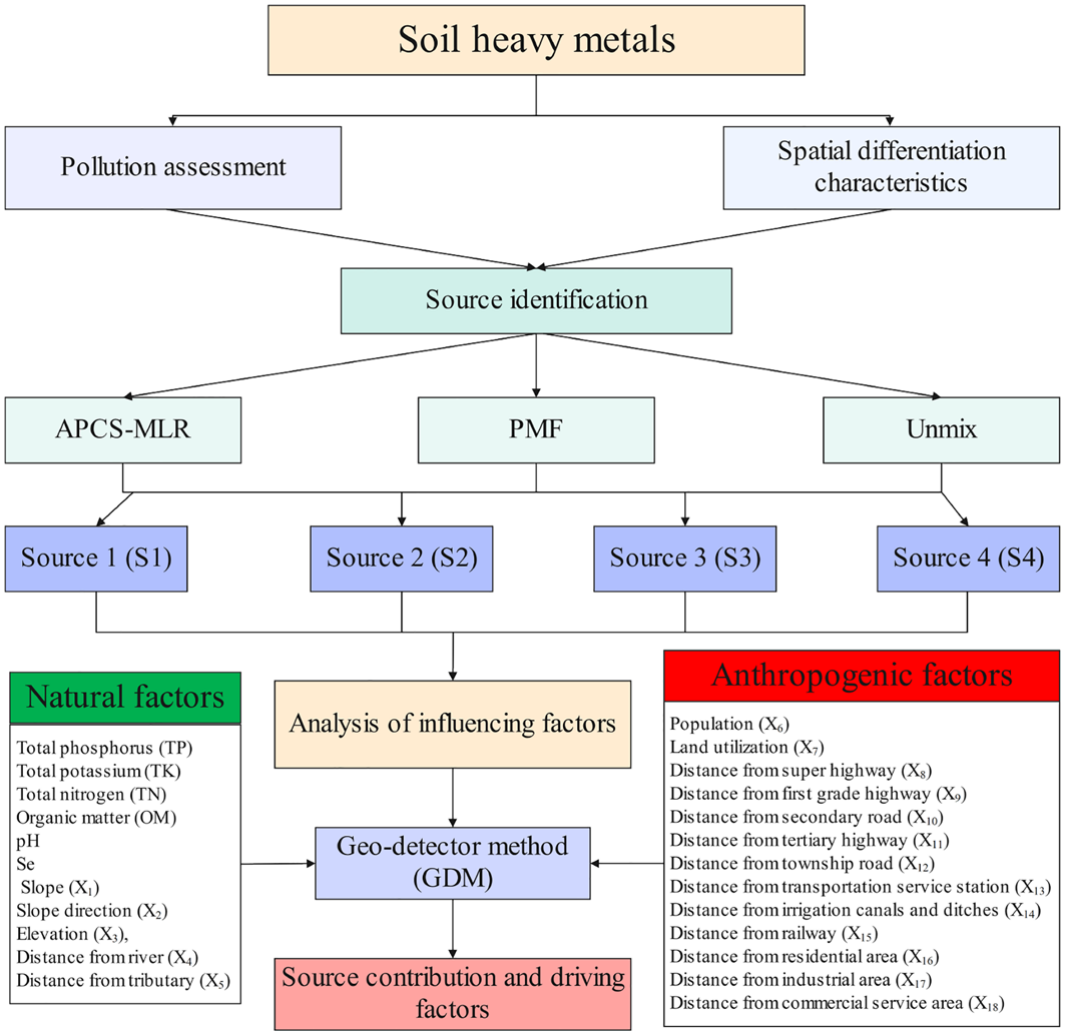
The schematic technical route of the comprehensive methodology.

The dependent variables are the mean factor scores of APCS-MLR and PMF, 24 explanatory variables including TP, TK, TN, OM, pH, Se, slope (X_1_), slope direction (X_2_), elevation (X_3_), distance from river (X_4_), distance from tributary (X_5_), population (X_6_), land utilization (X_7_),distance from super highway (X_8_), distance from first grade highway (X_9_),distance from secondary road (X_10_), distance from tertiary highway (X_11_), distance from township road (X_12_), distance from transportation service station (X_13_), distance from irrigation canals and ditches (X_14_), distance from railway (X_15_), distance from residential area (X_16_), distance from industrial zone (X_17_) and distance from commercial service area (X_18_) were selected. Natural factors are represented by TP, TK, TN, OM, pH, Se, X_1_–X_5_. Anthropogenic factors are represented by X_6_–X_18_. If the independent variable is numerical quantity, it needs to be discretized into type quantity^17^. The natural breakpoint method is used to divide 15 influencing factors into 10 categories. GeoDetector (http://www.geodetector.org/) SPSS26.0, ArcGIS10.8 and Origin2019 were used in this study. The schematic technical route of combining receptor models APCS-MLR, UNMIX and PMF with GDM was proposed in this study was showed in Fig. 2

## Results and discussion

### Concentrations and distribution of soil PTEs

The descriptive statistical characteristics of As, Cd, Cu, Cr, Hg, Ni, Pb and Zn concentrations in the overall soil and different land use types soils are showed in Table 1. Except for As, the concentration of total soil PTEs in study area was higher than the background value of Sichuan province (BSS)^28^, and the average values of Cu, Pb, Zn, Cr, Ni, Cd and Hg in soil were 1.13, 1.05, 1.26, 1.16, 1.14, 2.78 and 2.46 of the corresponding BSS, respectively. In particular, the maximum concentrations of Cu, Zn, Cd and Hg were 19.5, 21.0, 10.5 and 14.4 times of the corresponding BSS. The average values of Cu, Pb, Zn, Cr, Ni, Cd, As and Hg in cultivated soil were 1.15, 1.04, 1.28, 1.17, 1.14, 2.78, 0.90 and 2.62 times higher than their corresponding BSS, respectively. The average values of Cu, Pb, Zn, Cr, Ni, Cd, As and Hg in forest soil were 1.09, 1.06, 1.21, 1.15, 1.13, 2.66, 0.92 and 2.46 times higher than corresponding BSS, respectively. For other land soil, mean values of Cu, Pb, Zn, Cr, Ni, Cd, As and Hg were 1.15, 1.00, 1.23, 1.19, 1.10, 3.04, 0.94 and 3.11 times higher than corresponding BSS, respectively. In this study, the average values of soil PTEs were all lower than the risk screening value of soil PTEs (GB15618-2018)^10^, indicating that soil PTEs did not pose a significant threat to crops and humans on the whole. However, 1.52%, 0.13%, 0.25%, 0.13% and 13.1% of soil Cu, Pb, Zn, Cr and Cd contents exceeded the risk screening values, indicating that there was a significant risk of point source pollution in the study area.

**Table 1:** Descriptive statistics of soil PTEs in the study area.

		Cu	Pb	Zn	Cr	Ni	Cd	As	Hg
Total soils (n = 788)	Minimum (mg/kg)	18.5	19.8	55.0	61.7	23.6	0.082	4.17	0.022
	Maximum (mg/kg)	607	90.3	1820	264	56.6	0.83	18.0	0.88
	Median (mg/kg)	34.4	32.3	106	92.3	37.3	0.21	9.43	0.13
	Average mean (mg/kg)	35.2	32.3	108.9	91.9	37.1	0.22	9.76	0.15
	Standard deviation	21.3	5.01	63.5	10.1	3.75	0.07	2.09	0.09
	CV (%)	60.5	15.5	58.3	11.0	10.1	31.1	21.4	58.4
	Geometric mean (mg/kg)	34.2	31.9	105.9	91.5	36.9	0.21	9.55	0.14
Cultivated soils (n = 538)	Minimum (mg/kg)	18.5	19.8	55.0	61.7	26.7	0.082	4.17	0.030
	Maximum (mg/kg)	607	60.2	1820	133	56.6	0.83	18.0	0.71
	Median (mg/kg)	34.5	32.4	107	92.3	37.5	0.21	9.32	0.13
	Average mean (mg/kg)	35.7	32.2	110.6	92.1	37.3	0.22	9.67	0.16
	Standard deviation	25.2	4.55	76.1	7.94	3.67	0.07	2.02	0.08
	CV (%)	70.7	14.1	68.8	8.63	9.86	31.3	20.9	54.4
	Geometric mean (mg/kg)	34.4	31.9	107	91.7	37.1	0.21	9.47	0.14
Forest soils (n = 209)	Minimum (mg/kg)	19.0	20.4	59.8	64.0	23.6	0.092	4.93	0.038
	Maximum (mg/kg)	102	90.3	170	108	49.2	0.44	17.8	0.88
	Median (mg/kg)	34.4	32.4	106	92.5	36.9	0.21	9.52	0.13
	Average mean (mg/kg)	33.9	32.6	105	91.2	36.9	0.21	9.91	0.15
	Standard deviation	6.78	6.01	14.6	8.13	3.80	0.06	2.29	0.090
	CV (%)	20.0	18.4	13.9	8.92	10.3	28.6	23.1	61.2
	Geometric mean (mg/kg)	33.4	32.2	104	90.8	36.7	0.21	9.67	0.13
Other land soils (n = 41)	Minimum (mg/kg)	20.0	22.7	71.6	75.2	28.2	0.11	7.09	0.022
	Maximum (mg/kg)	96.6	46.0	152	264	45.4	0.47	14.8	0.60
	Median (mg/kg)	34.3	30.5	105	89.5	36.0	0.23	10.2	0.12
	Average mean (mg/kg)	35.8	30.9	106	93.7	35.9	0.24	10.2	0.19
	Standard deviation	11.0	4.8	16.6	27.9	4.21	0.09	1.80	0.14
	CV (%)	30.7	15.6	15.7	29.8	11.7	36.6	17.7	77.3
	Geometric mean (mg/kg)	34.7	30.6	104	91.6	35.7	0.23	10.0	0.14
BSS		31.1	30.9	86.5	79	32.6	0.079	10.8	0.061
RSV		50	90	200	150	70	0.3	40	1.8

*CV* Coefficient of variation, *BSC* Background value of soil in Sichuan province^28^, *RSV* Risk screening values (GB15618-2018)^10^.

In addition, the coefficient of variation (CV) values of Cu, Hg and Zn were all more than 50%, especially the CV values of Hg in different land use types were all above 50%, indicating that there were great spatial differences of these soils PTEs^29^.

**Figure 3:**
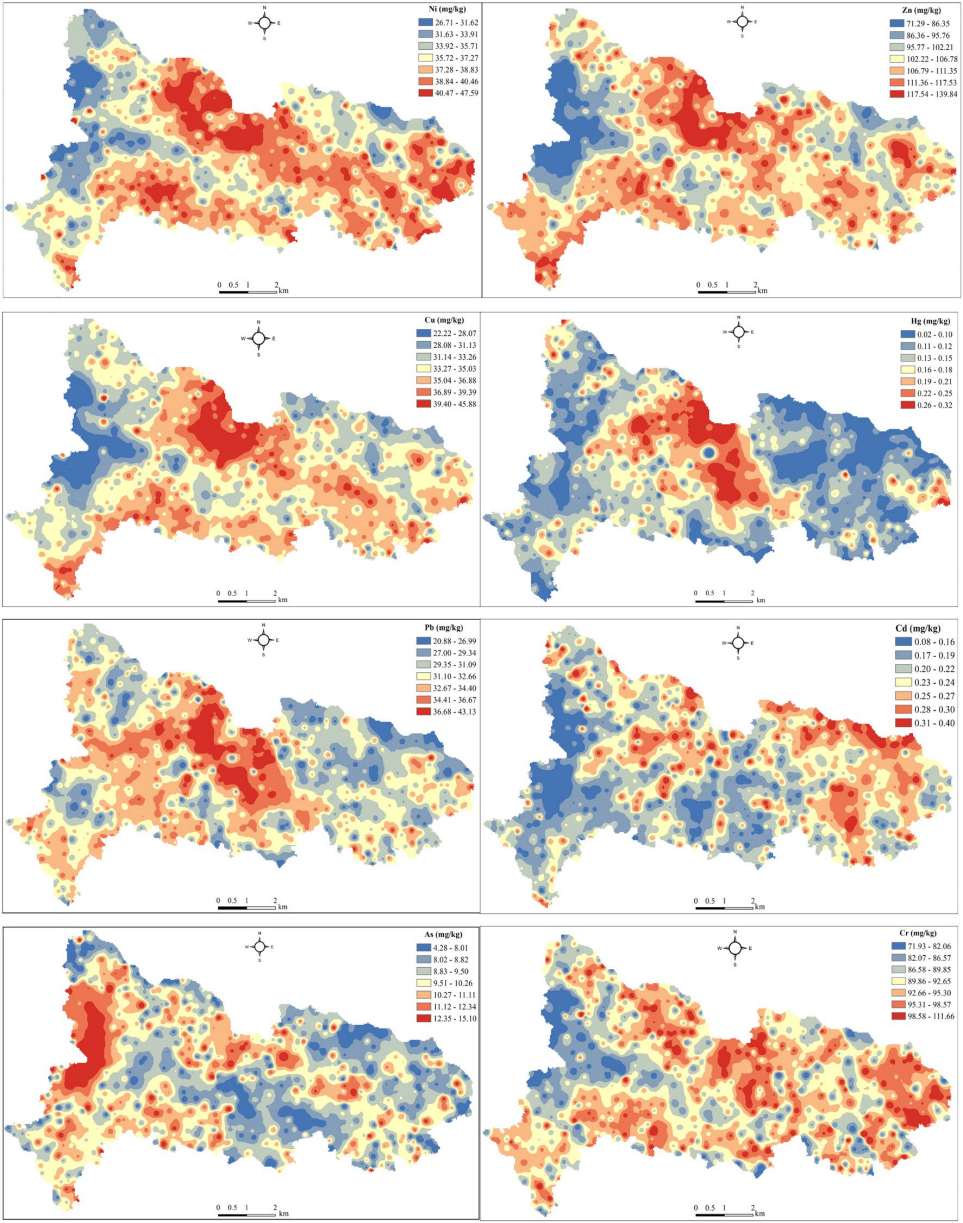
Spatial distribution of the soil PTEs (map were generated with software ArcMap10.8 http://www.esri.com/).

Figure 3 shows the spatial distribution of soil PTEs in the study area. It can be seen that the high value areas of Cu, Ni and Zn were generally distributed in the central and western regions, and the low content areas mainly appear in the western regions. The high content areas of Hg and Pb were mainly concentrated in the middle of study area. The high value areas of Cd were sporadically distributed in the central and eastern regions, and the eastern regions were generally higher than the central and western regions. The high value areas of As were mainly concentrated in the western regions, and the western regions were obviously higher than the central and eastern regions. The high value area of Cr was scattered in the whole study area. It can be seen that there is a significant difference in the spatial distribution of soil PTEs content on the town scale.

### Soil PTEs contamination level

**Figure 4:**
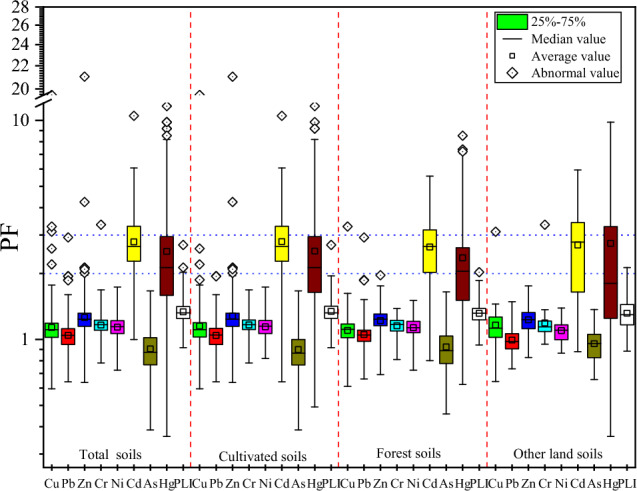
Pollution index of PTEs under total soil and different land utilization soil.

In order to better understand the level of soil PTEs pollution, we calculated the PF and PLI values of PTEs in total soils and different land use patterns soils in the study area (Fig. 4). The average PF values of PTEs in both the total soils and forest soils were in descending order: Cd > Hg > Zn > Cr > Ni > Cu > Pb > As, Cd > Hg > Zn > Cr > Cu > Ni > Pb > As in cultivated soils, and Hg > Cd > Zn > Cr > Cu > Ni > Pb > As in other soils. The average PF values of Hg and Cd in total soils and different use type soils were the highest, among which Hg and Cd in total soils, cultivated soils and forest soils were at 2–3 mild pollution levels, while in other use type soils were at 3–5 moderate pollution levels, indicating that soils were polluted by Hg and Cd to a certain extent under different land use types. The average PF values of Zn, Cr, Ni and Cu and Pb in the total soils and different utilization type soils were 1–2, which belong to slightly contaminated. The average PF value of As in all soils were less than 1, which belongs to the uncontaminated. The PLI value is calculated from the PFs of a single PTE to evaluate the pollution status of multiple PTEs. PLI values showed that total soils (1.34), cultivated soils (1.34), forest soils (1.32) and other soils (1.36) were moderately polluted. Overall, soils were contaminated with PTEs to varying degrees regardless of land use type. Overall, PF and PLI revealed that human activities influence the accumulation of soil PTEs. Human activities strongly affected Cd and Hg in the soil of the study area, followed by Zn, Cr, Ni, Cu and Pb, and had little effect on As. To understand the contribution of anthropogenic and natural activities to soil PTEs accumulation, a receptor model should be used to further quantify source resolution.

### Source apportionment of PTEs in soils

#### Evaluation of source apportionment of different receptor models

**Figure 5:**
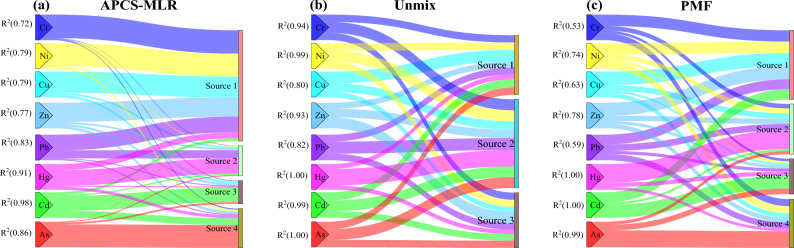
Contribution of each factor for PTEs in different receptor models.

Figure 5a–c, exhibits the composition of factors as analyzed by the APCS-MLR, UNMIX and PMF receptor models. The accuracies of the three models were evaluated by using the determination coefficient (R^2^) and their respective diagnostic parameters. The R^2^ values of each soil PTEs ranged from 0.72 to 0.98 in the APCS-MLR model, 0.80–1.00 in the UNMIX model, and 0.53–1.00 in the PMF model. As far as R^2^ was concerned, the Unmix model has the highest reliability, followed by APCS-MLR and PMF. In terms of their respective diagnostic parameters, the KMO and Bartlett test results of the APCS-MLRU model show that the KMO value was 0.771, meeting the requirement of > 0.6, and the *P* value was 0.000, meeting the requirement of *P* < 0.001^30^, which was suitable for APCS-MLR analysis (Fig. 4a). Previous studies have shown that APCS-MLR model source apportionment has good repeatability and produces relatively accurate results^31–33^. For the PMF model, the S/N of 8 kinds of soil PTEs were all greater than 7, indicating that the accuracy of the apportionment results was high (Fig. 4c), however, the R^2^ of Cr, Pb and Cu were 0.53, 0.59 and 0.63 respectively, all less than 0.7, which indicated that the fitness of these PTEs was not ideal. Previous studies have shown that the PMF model was extremely sensitive to outliers, and reasonable results can`t be obtained without eliminating outliers^15^. For the UNMIX model, the R^2^ of each soil PTEs was very high, and Min R^2^ was 0.94 and Min S/N was 2.10, which meets the requirement Min R^2^ > 0.8 and Min S/N > 2.0, respectively. UNMIX model can compensate for the loss of some elements contribution and is an effective tool for soil PTEs source analysis apportionment^16,34, 35^. Although the Unmix model identified three sources for all PTEs in this study, the proportion of each PTEs in the three sources was very close (Fig. 4b), which failed to effectively separate and identify the sources of each PTEs. This may be due to the relatively small change of soil PTEs in the small-scale areas of this study.

**Figure 6:**
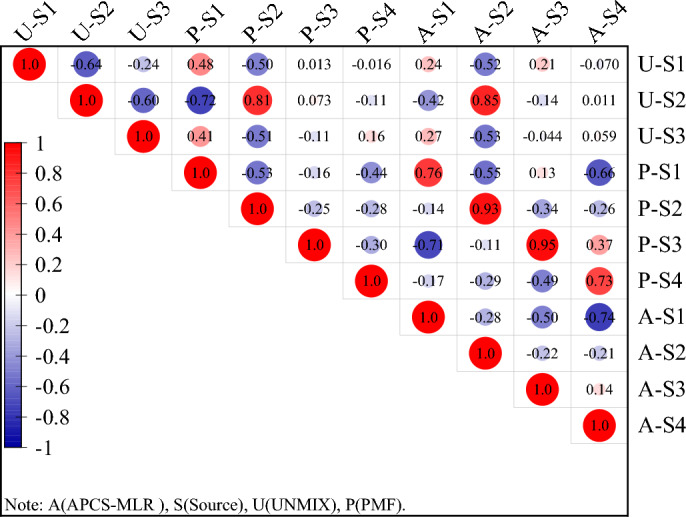
Pearson correlation showing contributions of factors derived from APCS-MLR, UNMIX and PMF.

Pearson correlation analysis was further used to explain the relationship between the factors obtained from the three models. As shown in Fig. 6, the same factors produced by the APCS-MLR model and the model PMF have a strong correlation. For example, the correlation coefficients of source 1 (S1), source 2 (S2), source 3 (S3) and source 4 (S4) in APCS-MLR and PMF were all greater than 0.80, reaching a significant level of 0.05. However, most of the factors produced by UNMIX have no correlation except the individual factors of APCS-MLR and PMF. In general, APCS-MLR model was considered to be the preferred model for soil PTEs quantitative source apportion in this study area.

#### Comparison of source apportionment results for different receptor models

In source apportionment, high load soil PTEs with certain factors can be served as typical PTEs. As shown in (Fig. 5a,c), S1 of APCS-MLR and PMF apportionment were mainly dominated by Cu, Pb, Zn, Cr and Ni, with contribution rates of 31.7–82.7%, 38.4–61.6%, 50.8–75.3%, 44.4–91.9% and 44.9–89.4%, respectively. S2 was mainly related to Hg, and the contribution rate of two models were 60.9–73.1%. In addition, Pb show moderate load with S2 in the two models, Cr and Ni show moderate load with S2 in PMF models. S3 of the two models were mainly related Cd, and the contribution rates was 50.9–57.7%. S4 has a significant correlation with As in two models, and the contribution rate was between 65.3 and 9.5%. Meanwhile, Cd shows moderate load with S4 in the APCS-MLR model, Pb, Cr and Ni show moderate load with S4 in the PMF model. As shown in Fig. 5b, S1 of UNMIX apportionment was mainly dominated by Cu. S2 was mainly related to Pb, Cr, Ni, Cd, As and Hg. S3 was mainly related to Zn. It is obvious that the results of UNMIX producing were quite different from those of APCS-MLR and PMF.

### Driving factors affecting pollution sources by GDM

**Figure 7:**
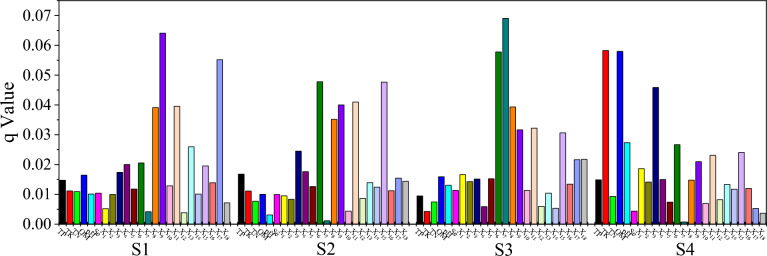
GDM results of 24 influence factors on 4 apportionment sources. X_1_: Slope; Slope X_2_: direction; X_3_: Elevation; X_4_: Distance from river; X_5_: Distance from tributary; X_6_: Population; X_7_: Land utilization; X_8_: Distance from super highway; X_9_: Distance from first grade highway; X_10_: Distance from secondary road; X_11_: Distance from tertiary highway; X_12_: Distance from township road; X_13_: Distance from transportation service station; X_14_: Distance from irrigation canals and ditches; X_15_: Distance from railway; X_16_: Distance from residential area; X_17_: Distance from industrial zone; X_18_: Distance from commercial service area.

Quantify the influence intensity of each influence factor on soil PTEs source apportionment factors by using the *q* value obtained from GDM. The maximum *q* value indicates that the influencing factor was the dominant explanatory variable of soil PTEs source apportionment fators^18,36, 37^. As shown in Fig. 7, S1 was dominated by X_9_ (q = 0.064, P < 0.01), followed by X_8_ (q = 0.039, P < 0.01), X_11_ (q = 0.064, P < 0.01) and X_17_ (q = 0.055, P < 0.01), indicating that S1 has a significant spatial correlation with X_8_, X_9_, X_11_ and X_17_, indicating that S1 represented the anthropogenic source of soil pollution. X_6_, X_8_, X_9_, X_11_ and X_15_ had significant spatial correlation with S2, with q values of 0.048 (P < 0.01), 0.035 (P < 0.05), 0.040 (P < 0.05) , 0.041 (P < 0.01) and 0.048 (P < 0.01), respectively. X_6_ and X_7_ have very significant spatial correlation with S3 (P < 0.01), and q values were 0.058 and 0.069, respectively, X_8_, X_9_, X_11_ and X_15_ have significant spatial correlation with S3 (P < 0.05), which means that S3 represents the anthropogenic source of soil pollution. TK (q = 0.058, P < 0.01), OM (q = 0.058, P < 0.01) and X_3_ (q = 0.046, P < 0.01) had very significant spatial correlation with S4, indicating that S4 represented the natural source of soil pollution.

### Interpretation of sources obtained by multiple methods

#### S1 interpretation

According to the multi-receptor model (Fig. 4), S1 contributes the most to Cu (82.7%), Pb (61.6%), Zn (75.3%), Cr (91.9%) and Ni (89.4%) in soil, followed by Hg (23.6%), Cd (10.6%) and As (0.3%). The results showed that S1 had the greatest contribution to Cu, Pb, Zn, Cr and Ni in soil, followed by Hg. In general, Cu, Pb and Zn may come from traffic emissions caused by motor vehicle engine, brake pads, galvanized parts and tires wear^5,38^. Previous studies have shown that Pb mainly comes from traffic emissions, such as leaded gasoline emissions, engine brake wear and catalytic combustion^39^, as well as Zn pollution caused by tire wear or galvanized parts corrosion^40^. It was believed that copper oxide catalysts were often used in engine combustion systems to control carbon monoxide emissions from automobile exhaust^41^. In addition, industrial activities, including hardware industry, metallurgical industry and electronics industry, will further increase the accumulation of soil Cd, Zn, Cu, Cr and Ni^42^. It was found that Cr in soil is related to industrial production activities such as wear of metal parts and chromium-plated parts, iron and steel production and metallurgical manufacturing^43^. Cr metal particles were produced in the operation and production process of iron and steel plants, hardware market, logistics base and automobile and parts industry, especially Cr elements will be released in different degrees in all aspects of cement production, which will lead to the enrichment of Cr in the surrounding soil^44^.

Based on the results of GDM, the spatial correlation between S1 with X_8_, X_9_, X_11_ and X_17_ were the highest. There are a large number of roads distributed in the study area, and the traffic and traffic flow are very busy. With the rapid increase of the number of motor vehicles in Chengdu, the traffic flow, road dust density and tail gas emission also increase sharply. potentially toxic element particles generated by road traffic activities are eventually enriched in the surrounding soil through atmospheric sedimentation and air dust adsorption. In addition, there were factories and enterprises in the study area, such as building materials decoration, plastic production, cement production, concrete manufacturing, red brick firing and printing, etc. The potentially toxic element particles and raw materials that may be scattered in these shops and workshops, as well as the PTEs carried by the “Three wastes” produced by industrial activities, were enriched into the soil by atmospheric sedimentation, rain water erosion and infiltration, causing the accumulation of soil PTEs. Overall, S1 represented traffic-industrial source.

#### S2 interpretation

S2 was dominantly explained by Hg (60.9%), followed by Pb (31.5%), whereas low loading were observed for Cu (7.1%), Zn (6.9%), Cr (1.4%), Ni (3.0%), Cd (4.5%) and As (3.6%). In this study, the high CV value (Table 1) and PF value (Fig. 3) of Hg indicate that human activities strongly influence the accumulation of soil Hg. Relevant research shows that Hg was related to coal combustion^45,46^, waste gas emission^47,48^ and mineral mining, and these industrial activities will increase the emission of Hg into the atmosphere. In addition, some studies show that traffic factors have a great effect on the accumulation of Hg^49^, and the exhaust gas of gas companies will also emit Hg into the atmosphere^50^. Hg in soil was mostly from the dry and wet deposition of the atmosphere^51^. It has been reported that, railway transportation as a serious source of organic and inorganic pollution^52,53^.

GDM shows that X_6_ and X_15_ were the highest driver of S2 (Fig. 7), followed by X_8_, X_9_, X_11_. It means that human domestic emissions and traffic activities have the greatest impact on S2. PTEs produced by human production, domestic and transportation activities can enter the soil through atmospheric deposition, and then transfer to other places through runoff. In addition, as shown in Fig. 3, the hot spots of Hg′s spatial distribution appear in the central area with heavy traffic and dense population. Therefore, S2 can represent domestic-transport emissions source.

#### S3 interpretation

The content of Cd (57.7%) was the highest in S3, and the content of other PTEs were less than 10% in S3. Under land utilization, the PF value of soil Cd in the study area was the highest (Fig. 4), reached the level of mild and moderate pollution. Previous studies have shown that Cd has been introduced into soil with the agricultural application of phosphate fertilizers, organic fertilizers and pesticides^54^. In addition, a large amount of Cd was discharged into the environmental medium through waste water, waste gas and waste residue, and then into the soil^55^.

The results of GMD showed that there was a significant spatial correlation between S3 with X_6_ and X_7_, indicating that agriculture activity was the main driving factor of S3. It has been reported that phosphorus fertilizer was the main source of Cd in French farmland soil, accounting for more than 1/2 of the input of Cd in French agricultural soil^56^. The input flux of Cd through fertilization in Songnen plain soil in China ranges from 0.001 to 0.255 mg/m^2^/a^57^, and the input flux of Cd through insecticides (oxazepine, chlorpyrifos and carbendazam) in Zhangshi Irrigation Area of Shenyang was 2.0 × 10^−6^ mg/m^2^/a^58^. According to our field investigation, in order to increase yield, people increased the use of pesticides, chemical fertilizers and organic fertilizers, thus bringing Cd into the soil and increasing the accumulation of soil Cd. Therefore, S3 can represent the agricultural source.

#### S4 interpretation

The explanation of S4 was mainly As (89.5%). In this study, the average value of As in the soil of the whole region was 0.90 times of its corresponding background value, indicating that the impact of human activities was limited (Table 1). In addition, the mean PF value of As showed relatively low pollution (Fig. 4), which confirmed that the concentration of soil As in this study area was dependent on natural sources. Previous studies have reported that As in soil may be an indicator of soil parent material^59^. However, anthropogenic input of As includes agricultural activities, such as the application of organic and chemical fertilizers and sewage irrigation^60^, and industrial emissions^61,62^.

Combined with GMD results (Fig. 6), S4 had the highest spatial correlation with TK (q = 0.058, P < 0.01), OM (q = 0.058, P < 0.01) and X_3_ (q = 0.046, P < 0.05), and TK, OM and X_3_ represents the natural influencing factors. Therefore, S4 can represent the natural source.

### Significance of multi-source interpretation

On the one hand, since soil PTEs comes from both natural sources and human activities such as agriculture, transportation, and industry, PF, PLI, geoaccumulation index and other methods can only quantify the pollution level of soil PTEs, but can’t quantify the contribution of soil PTEs. On the other hand, the source of soil PTEs in a given region is not fixed and will change over time, and the emission intensity of different sources will also be different. In this case, the receptor model is very useful for analysis the source of soil PTEs, especially in the absence of detailed information about the source of emissions. However, use receptor models to identify source of soil PTEs must be interpreted based on previous research experience. However, the combination of receptor models (APCS-MLR, PMF, UNMIX) and GMD can solve this puzzle and explain the origin of soil PTEs more effectively.

In order to further explain the source factors from the receptor model, this study considers the influencing factors such as soil TP, TK, TN, OM, pH, elevation, slope, and distance from the sampling point to the river as natural pollution sources; the influencing factors such as the distance from the sampling point to industrial land and road as industrial pollution sources and traffic pollution sources; the distance from the sampling point to the residential land and the number of population were considered as domestic pollution sources; land use was considered as agricultural pollution sources. GMD was used to identify the main influencing factors of soil PTEs source factors. GMD results can effectively define the source factors obtained by the receptor model, which specific pollution sources were natural, industrial pollution, traffic, domestic, agricultural and other pollution sources, or were affected by multiple pollution sources together. Overall, this integrated approach provides a deep understanding of the multiple sources of soil PTEs pollution and a better definition of pollution factors.

## Conclusions

With the rapid development of industry and agriculture, PTEs pollution in farmland soil has become increasingly serious. In this study, receptor model and GDM were combined to quantitatively analyze the driving factors of soil PTEs in the central zone of Chengdu Plain, Sichuan Province. Except As, the PTEs content in soil was higher than the corresponding soil background value in Sichuan Province. APCS-MLR model can fit the observed and predicted PTEs values of most soils in the study area well, and was an ideal receptor model. The APCS-MLR model was used to identify four potential pollution sources, including traffic-industrial pollution source S1 (Cu (82.7%), Pb (61.6%), Zn (75.3%), Cr (91.9%) and Ni (89.4%)), domestic-transportation pollution source S2 (Hg (60.9%)), agricultural sources S3 (Cd (57.7%)) and natural sources S4 (As (89.5%)). GDM results showed that Distance from first grade highway, population, land utilization, and TK content contributed the most to S1, S2, S3, and S4, which helped explain the source factors derived from the receptor model.

In fact, soil potentially toxic element accumulation was a complex process, which was affected by many environmental factors. In the next research, more environmental factors affecting soil PTEs accumulation should be considered, such as precipitation, Gross Domestic Product (GDP), fertilizer amount, soil parent material, etc., and the spatial correlation between the influencing factors and the source factors should be deeply analyzed. At the same time, a comprehensive study and analysis of inventory data such as traffic emissions, fertilizer consumption, industrial emissions, and atmospheric subsidence were carried out to verify the results of the combination of receptor model and GDM.

## Data Availability

The authors declare that all data supporting the findings of this study are available within the article.
